# Clinical Care Guideline Implementation of Nebulized Tranexamic Acid in Post‐Tonsillectomy Hemorrhage

**DOI:** 10.1002/lary.70641

**Published:** 2026-05-29

**Authors:** Jennifer Lavin, Kathleen Billings, Alyssa Smith, Krupa Patel, Jaqueline Corboy, Inbal Hazkani

**Affiliations:** ^1^ Divison of Pediatric Otolaryngology Ann & Robert H. Lurie Children's Hospital of Chicago Chicago Illinois USA; ^2^ Department of Otolaryngology‐Head and Neck Surgery Northwestern University Feinberg School of Medicine Chicago Illinois USA; ^3^ Department of Otolaryngology‐Head and Neck Surgery Stanford University School of Medicine Stanford California USA; ^4^ Division of Pediatric Emergency Medicine Ann & Robert H. Lurie Children's Hospital of Chicago Chicago Illinois USA; ^5^ Department of Pediatrics Northwestern University Feinberg School of Medicine Chicago Illinois USA

**Keywords:** adenotonsillectomy, post‐tonsillectomy bleeding, post‐tonsillectomy hemorrhage, quality improvement, tranexamic acid, TXA

## Abstract

**Objectives:**

Nebulized tranexamic acid (TXA) can be utilized for non‐operative management in post‐tonsillectomy hemorrhage (PTH). Rapid adoption of new treatments in hospital settings is challenging due to personnel turnover, formulary restrictions, and inconsistent awareness. We investigated the utility of clinical care guideline (CCG) implementation on adherence to a TXA protocol and its association with operative control of hemorrhage.

**Methods:**

Model for Improvement methodology was utilized to develop CCG for TXA administration. An algorithm was created where patients presenting to the Emergency Department (ED) with active bleeding or blood clot received three nebulized TXA treatments. Exclusion criteria were severe bleeding, absence of active bleeding or clot, and inability to tolerate treatment or protect the airway. An order set was used to facilitate implementation. Data from 2 years pre‐ and post‐initiation were compared. Measures included ED returns for PTH, TXA order set usage, TXA administration frequency, returns to the operating room, and secondary returns to the ED.

**Results:**

There were 2805 and 5382 tonsillectomies pre‐ and post‐ implementation periods respectively. There was no difference in patient age in the two groups. ED returns for bleeding were 70 (2.5%) and 155 (2.9%) respectively (*p* > 0.05). Post‐implementation, 126 patients met inclusion criteria for TXA (81.3%). Order set utilization of patients receiving TXA in the ED was 95.7%. Operative PTH management pre‐ and post‐intervention was 35/70 (50%) and 42/155 (27.1%) respectively (*p* = 0.001, ARR 0.320, 95% CI 0.116–0.500).

**Conclusions:**

Implementation of a PTH clinical care guideline was associated with rapid adoption of TXA adherence. This was associated with reduced rates of OR returns.

**Level of Evidence:**

3

## Graphical abstract

In our quality initiative, we demonstrated how clinical care guidelines can be used as a strategy to implement tranexamic acid treatment of post‐tonsillectomy hemorrhage. High rates of algorithm adherence were observed with an associated reduction in need for operative control of hemorrhage.

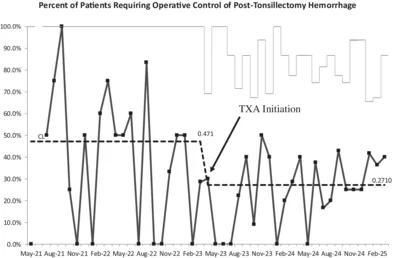

## 1 Introduction

With 567,000 procedures being performed in 2023, adenotonsillectomy (T&A) is the most common surgical procedure performed on children in the United States [1]. It is estimated that 1.1%–4.75% of surgeries are complicated by post‐tonsillectomy hemorrhage (PTH), making it the biggest source of postoperative morbidity in these patients [2]. Furthermore, current first‐line treatment using non‐steroidal anti‐inflammatory drugs may be associated with increased PTH [3, 4, 5].

Patients presenting to the Emergency Department (ED) with PTH have historically been managed by either observation or return to the operating room for hemostasis. Due to emerging data on the use of inhaled tranexamic acid (TXA) on pulmonary hemorrhage, there has been increasing interest in treating patients presenting with PTH with nebulized TXA as a means of avoiding surgical intervention [6]. Meta‐analyses have suggested that prophylactic intravenous TXA administration is associated with reduced blood loss in children after T&A, and nebulized TXA is a safe and promising treatment for PTH when used in the ED [7, 8].

As with any emerging treatment, rapid and uniform adoption of new protocols is challenging. In the setting of PTH, challenges are exacerbated by the need for co‐management of patients between teams in the ED and otolaryngology by introducing additional stakeholders who must agree with the proposed treatment. Additionally, at some centers, new therapeutic treatments require pharmacy approval prior to initiation, creating additional barriers to rapid change. At our institution, there was no established pathway for treating PTH patients with nebulized TXA, and this preparation was not available within our hospital's formulary, potentially increasing a patient's risk for more invasive management strategies.

We have previously demonstrated that clinical care guidelines (CCG)—with standard algorithms, order sets, patient instructions and note templates—have facilitated rapid standardization of care with high rates of adherence to protocols across our pediatric otolaryngology division [9, 10, 11]. To date, our team has only used similar strategies to standardize care that is well‐established. We hypothesized that this same quality improvement implementation strategy could be used to rapidly and consistently adopt a newer treatment, nebulized tranexamic acid, for the management of post‐tonsillectomy hemorrhage. Doing so will permit teams to nimbly adopt new treatments in a uniform way so that patients receive the most up‐to‐date care.

In our improvement initiative, we aimed to create and implement a CCG for patients presenting to the ED with PTH, where qualifying patients received nebulized TXA upon arrival to begin by April 1, 2023. In doing so, we aimed to treat 50% of qualifying patients with TXA with resultant reduction in need for operative hemostasis by 25% by August 2023.

## 2 Methods

### 2.1 Ethical Considerations

This work was reviewed by the Lurie Children's Hospital Institutional Review Board (#2022‐5591) and was approved.

### 2.2 Context

Our institution is an urban, free‐standing 364‐bed pediatric academic tertiary care center with two surgical satellites with T&A's being performed at all the operative sites. The Division of Otorhinolaryngology is composed of 14 surgeons, 2 fellows, and rotating residents who spend 2–6 months on the service. The ED is staffed by attending pediatric emergency medicine physicians, fellows, and rotating trainees. Prior to the outset of the improvement initiative, nebulized TXA was not an approved medication by the hospital pharmacy, and there was no process for administration.

### 2.3 Interventions

**FIGURE 1:**
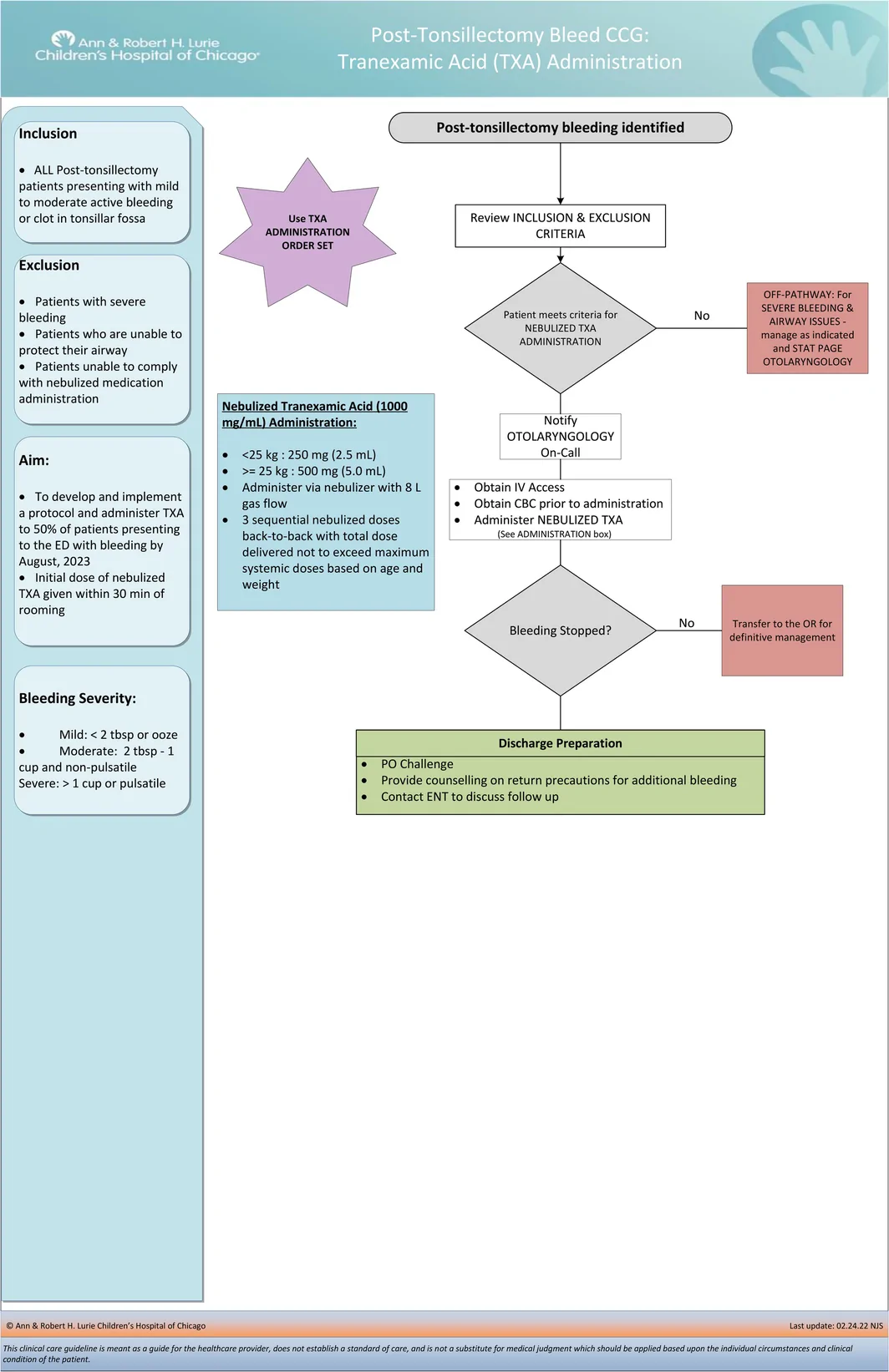
Algorithm for administration of tranexamic acid (TXA) in patients presenting to the Emergency Department for post‐tonsillectomy hemorrhage. [Color figure can be viewed in the online issue, which is available at www.laryngoscope.com]

The project team, consisting of representatives from otolaryngology, emergency medicine, and pharmacy and therapeutics, designed an algorithm for patients presenting to the ED with PTH (Figure 1). In the algorithm, inclusion criteria included patients with mild to moderate active bleeding (less than one cup and non‐pulsatile) or blood clot in the tonsillar fossa. Exclusion criteria in patients with active bleeding included severe bleeding (defined as more than one cup or pulsatile), inability to manage airway, and inability to tolerate TXA administration. Patients without active bleeding at time of evaluation were also excluded from treatment. Included patients were treated with three doses of nebulized TXA [12]. If no active bleeding was noted at the completion of treatment, patients were given an oral challenge and discharged when appropriate. Of note, during the initial phases of the intervention, patients were observed overnight to ensure safety. After several months, same‐day discharges were then permitted. Patients with persistent active hemorrhage were taken to the operating room for control. Patients were excluded from treatment if they presented with primary PTH, or with secondary PTH without active bleeding or clot, with severe hemorrhage, or with inability to protect their airway or tolerate the nebulizer.

To operationalize the algorithm, pharmacy approval was obtained for nebulized TXA administration. An order set was created with appropriate weight‐based dosing. A link to the algorithm was embedded into the order set and nebulized TXA could only be ordered via the established order set to minimize deviations from the algorithm. The algorithm and order set were implemented on April 4, 2023 without a wash‐in period. Prior to implementation, nurses and physicians in the ED and in otolaryngology received formal live and written education on the new algorithm, order set availability, and the technique of TXA administration.

Patients presenting to the ED with PTH between April 1, 2021 and March 31, 2025 were tracked. Outcome measures included percentage of patients requiring operative control of PTH. To perform study of intervention, process measures tracked included order set utilization rate and rate of TXA administration. Balancing measures included 30‐day all‐cause returns to the ED after discharge. Interventions were studied both via order set utilization and through feedback from frontline providers.

Statistical analysis was performed using *t*‐tests for continuous variables and chi‐square tests for categorical variables as appropriate (Graphpad, Boston, MA). Statistical process control analysis using control charts was used to track temporal trends in order set utilization and returns to operating room, with standard definitions for centerline shifts being employed.

## 3 Results

A total of 2805 and 5382 patients underwent T&A during the pre‐ and post‐intervention periods respectively. There was no statistical difference in age between the two groups, but there were statistically more males who underwent T&A in the post‐intervention period, and racial demographics and payer mix varied slightly (Table 1). Pre‐intervention, 70 patients (2.5%) presented to the ED with PTH compared to 155 patients (2.9%) post‐intervention (*p* > 0.05, 95% CI −0.003 to 0.01).

**TABLE 1:** Demographics of pre‐ and post‐intervention patients.

	Pre‐intervention	Post‐intervention	*p*
Average age (years)	6.5	6.6	> 0.05
Race, *n* (%)			**0.0002**
White	894 (31.9)	1958 (36.4)	
Black/African American	338 (12.0)	558 (10.4)	
Hispanic/Latino	1111 (39.6)	2095 (38.9)	
Asian	76 (2.7)	140 (2.6)	
Other	386 (13.8)	631 (11.7)	
Payer mix, *n* (%)			**0.002**
Commercial	1711 (61.0)	3068 (57.0)	
Medicaid	1066 (38.0)	2260 (42.0)	
Other	28 (1.0)	54 (1.0)	
Sex, *n* (%)			
Male	1303 (53.5)	3006 (55.8)	
Female	1502 (46.5)	2376 (44.2)	
BMI	21.3	19.8	**< 0.0001**

*Note:* Bold emphasis designates values that are statistically significant.

In the post‐intervention period, 126 of the patients presenting to the ED with PTH met criteria for TXA (81.3%) with 115 patients receiving treatments in our ED, and 11 receiving them at referring hospitals prior to transfer based on the guidance of our hospital at time of transfer acceptance. All patients who met criteria for TXA received treatment. Of patients treated with TXA in our ED, the order set was utilized in all but five encounters (95.7%). The most common reason for exclusion of TXA was absence of active bleeding or clot (Table 2).

**TABLE 2:** Reasons for exclusion from TXA treatment.

	*n* (%)
No active bleeding	26 (89.7)
Did not tolerate	2 (6.9)
Primary hemorrhage	1 (3.4)

Abbreviation: TXA, tranexamic acid.

**FIGURE 2:**
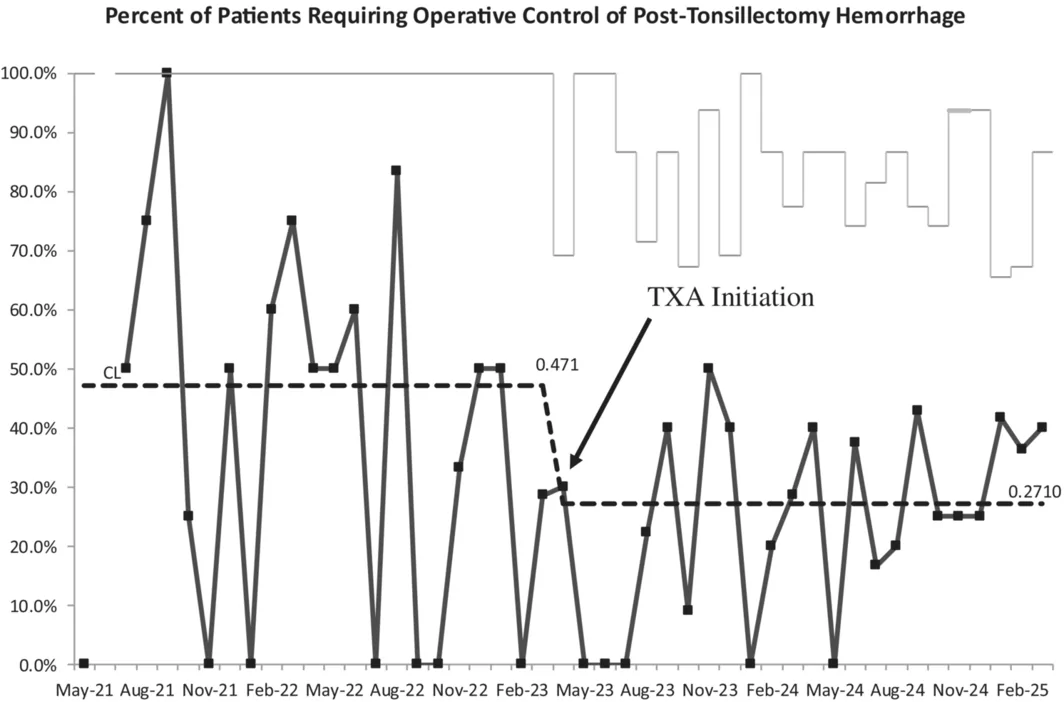
P‐chart of monthly percentage of patients returning to the Emergency Department for post‐tonsillectomy hemorrhage who require operative management of bleeding. UCL–upper control limit.

Prior to TXA initiation, 35/70 patients returning to the ED for bleeding were taken to the OR for surgical control (50%), compared to 42/155 (27.1%) post‐intervention (*p* = 0.001 ARR 0.320, 95% CI 0.116–0.500). Of patients treated with TXA, 40/126 (31.7%) required operative hemostasis after nebulizer therapy. Control chart analysis demonstrated a centerline shift that coincided with the intervention (Figure 2). Secondary returns to the ED after discharge were 8/70 (11.4%), and 11/155 (7.1%) pre‐ and post‐intervention respectively (*p* = 0.3). Furthermore, no statistical difference was observed in secondary return rates in patients treated with TXA versus not treated with TXA in the post‐intervention period.

## 4 Discussion

Due to the high number of tonsillectomies performed in children, PTH represents a significant cause of morbidity and is a frequent reason for unanticipated returns to the operating room in pediatric patients. Such interventions subject patients to additional anesthetic risks and extend the duration of analgesic requirements. In recent years, TXA has emerged as a non‐invasive strategy to treat PTH, and studies have previously demonstrated associated reductions in need for operative control [7, 8, 13, 14]. Widespread implementation of PTH management with TXA has the potential to greatly reduce patient morbidity as well as alleviate the burden on often overextended operating rooms.

Rapid adoption of new treatments is challenging. Barriers to success include but are not limited to lack of awareness, hesitancy to modify established practice, uncertainty of appropriateness criteria and regimen, and logistical concerns. As such, new treatments are often gradually adopted. In the studies by Spencer et al. and Shin et al., for example, only a subset of patients presenting with active bleeding received TXA, with indications depending on provider preference rather than inclusion criteria [13, 14].

In our improvement initiative, a CCG was implemented that included an algorithm paired with standardized order sets for treatment. Upon initiation, we observed rapid adoption of change in practice with 100% of patients meeting inclusion criteria receiving TXA in the ED. While not proven, it is suspected that high rates of adherence were facilitated through such a standardized approach to implementation. At our institution, the presence of numerous pediatric otolaryngologists and emergency medicine providers, along with rotating trainees from multiple outside institutions, presents challenges to consistent implementation. Many of these providers may be unfamiliar with tranexamic acid (TXA) as a therapeutic option. In addition, pharmacists must authorize its use, and respiratory therapists must be well‐versed in its administration. Without a structured, standardized approach, efforts to introduce TXA risk poor adherence and inconsistent uptake across disciplines.

TXA administration was intended to be standardized through dedicated order sets, with the electronic medical record configured to prevent ordering outside of this pathway. Despite this, five patients received TXA without use of the order set. The mechanism for this workaround is unclear. Given the rarity of these occurrences and lack of persistence over time, no targeted mitigation strategies have been implemented.

We have previously reported on the use of CCGs in the management of post‐T&A pain control, and in patients presenting with coin‐shaped esophageal foreign bodies [9, 10, 11]. All previously described CCG efforts have been aimed at standardizing care with well‐established diagnosis and treatment protocols. In this current initiative, we have demonstrated that similar strategies can be utilized to rapidly adopt novel treatments hospital‐wide, suggesting a means of implementing future new diagnostic and treatment strategies.

While causation cannot be proven, after implementation of the new TXA protocol, our hospital observed a 45.8% reduction in need for operative control in patients returning to the ED for PTH. Control chart analysis suggests a temporal association between the intervention and an observed reduction of operative control. It is notable that observer bias may contribute to this shift; however, as surgeons may be more comfortable observing a patient with a small clot in the tonsillar fossa if TXA has been administered. Additional prospective, blinded studies would be helpful in further elucidating the true effects of TXA administration in this population.

One concern with treating patients with TXA may be the potential for an increase in secondary ED returns should the therapeutic effect not be sustained. In our study, the secondary return rate was not different in the post‐intervention period. Tracking balancing measures is essential to quality improvement to ensure there are no unintended consequences of an intervention.

Comparison of the pre‐ and post‐intervention cohorts demonstrated a significant increase in the number of T&A procedures performed. Similar increases in surgical volume have been anecdotally reported across pediatric hospitals in the immediate post–COVID‐19 period. Despite the increase in surgical volume, the age distribution of patients remained similar between cohorts. Given that age is associated with variation in hemorrhage rates, this stability in distribution is notable [15].

Although not formally assessed, feedback regarding the CCG was generally positive. In the context of increasing healthcare worker burnout, interventions that reduce the need for invasive procedures may lessen strain on already overextended operating rooms. Moreover, the potential for fewer additional procedures—thereby shortening recovery times and decreasing reliance on prolonged analgesic therapies—underscores the patient‐centered benefits of this approach. Collectively, these findings highlight both the clinical and operational value of implementing such a care guideline.

Limitations of this study include lack of data on comorbidities between cohorts. Similarly, due to large sample size, the exact technique for each tonsillectomy is unknown, representing a source of potential confounding variables. Given that post‐tonsillectomy hemorrhage rates remained stable, it is not suspected to represent a large confounder. Similarly, the exact amount of hemorrhage was not quantified in this study, introducing another potential source of confounders.

## 5 Conclusions

Through our improvement initiative we utilized a CCG to rapidly adopt the use of TXA in patients presenting to the ED with post‐tonsillectomy hemorrhage. This approach resulted in 100% of eligible patients receiving TXA in the ED and was associated with a significant reduction in the need for operative control of bleeding. More broadly, our findings illustrate that CCGs may serve not only as vehicles for implementing established therapies but also as frameworks for the rapid adoption of emerging treatments. Additional study of applicability in other institutions would further elucidate the generalizability of our findings. If this is found to be consistent in other settings, institutions can integrate new interventions more efficiently, circumventing the traditional adoption curve and potentially accelerating improvements in patient outcomes.

## Funding

The authors have nothing to report.

## Conflicts of Interest

The authors declare no conflicts of interest.

## Data Availability

The data that support the findings of this study are available on request from the corresponding author. The data are not publicly available due to privacy or ethical restrictions.
